# Hepatitis E Virus: How It Escapes Host Innate Immunity

**DOI:** 10.3390/vaccines8030422

**Published:** 2020-07-28

**Authors:** Sébastien Lhomme, Marion Migueres, Florence Abravanel, Olivier Marion, Nassim Kamar, Jacques Izopet

**Affiliations:** 1 National Reference Center for Hepatitis E Virus, Toulouse Purpan University Hospital, 31300 Toulouse, France; migueres.m@chu-toulouse.fr (M.M.); abravanel.f@chu-toulouse.fr (F.A.); izopet.j@chu-toulouse.fr (J.I.); 2 INSERM UMR1043, CNRS UMR5282, Center for Pathophysiology of Toulouse Purpan, 31300 Toulouse, France; marion.o@chu-toulouse.fr (O.M.); kamar.n@chu-toulouse.fr (N.K.); 3 Université Toulouse III Paul Sabatier, 31330 Toulouse, France; 4 Department of Nephrology and Organs Transplantation, Toulouse Rangueil University Hospital, 31400 Toulouse, France

**Keywords:** hepatitis E virus, innate immune response, immune escape

## Abstract

Hepatitis E virus (HEV) is a leading cause of viral hepatitis in the world. It is usually responsible for acute hepatitis, but can lead to a chronic infection in immunocompromised patients. The host’s innate immune response is the first line of defense against a virus infection; there is growing evidence that HEV RNA is recognized by toll-like receptors (TLRs) and retinoic acid-inducible gene I (RIG-I)-like receptors (RLRs), leading to interferon (IFN) production. The IFNs activate interferon-stimulated genes (ISGs) to limit HEV replication and spread. HEV has developed strategies to counteract this antiviral response, by limiting IFN induction and signaling. This review summarizes the advances in our knowledge of intracellular pathogen recognition, interferon and inflammatory response, and the role of virus protein in immune evasion.

## 1. Introduction

Hepatitis E Virus (HEV) is the major cause of viral hepatitis worldwide, with an estimated 20 million infections annually, including about 3.3 million cases of symptomatic hepatitis [1]. HEV infections are usually self-limiting, but can lead to acute liver failure in pregnant women living in developing countries [2,3]. The virus also causes extra-hepatic manifestations, especially renal and neurological disorders [4,5]. It can result in chronic infections in immunocompromised patients, such as solid organ transplant recipients and patients with an HIV infection or hematological diseases [3]. A chronic infection is defined as the persistence of HEV RNA in a patient’s samples for more than 3 months [6].

HEV belongs to the *Hepeviridae* family, a family that has two genera: the *Orthohepevirus* (mammalian and avian strains), with four species (*A*–*D*), and the *Piscihepevirus* (cutthroat trout virus) [7,8]. Humans are infected by *Orthohepevirus A,* although several cases of *Orthohepevirus C* infection have been reported recently [9,10,11]. *Orthohepevirus A* includes at least eight distinct genotypes (HEV1–8). Some infect humans (HEV1, -2, -3, -4, and -7) or pigs (HEV3 and -4), wild boar (HEV3, -4, -5, and -6), rabbits (HEV3), mongooses (HEV3), deer (HEV3), yaks (HEV4), and even camels (HEV7 and HEV8). Two genotypes have been described in *Orthohepevirus C* to date. HEV-C1 infects hosts belonging to the orders Rodentia and Soricomorpha, and HEV-C2 infects hosts of the order Carnivora [12].

**Figure 1:**
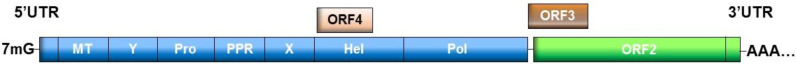
Organization of hepatitis E virus genome. ORF1 (dark blue box) encodes the nonstructural polyprotein. ORF4 has only been detected in HEV1. 7 mG: 7-methylguanosine cap; AAA…: polyadenylated tail; Hel: helicase; MT: methyltransferase; Pol: RNA polymerase; PPR: polyproline region, also called the hypervariable region; Pro: cysteine protease; UTR: untranslated region; X: X domain or macrodomain; Y: Y domain.

HEV is a small virus with a positive-sense, single-stranded, ~7.2 kb RNA genome. The *Orthohepevirus A* genome contains three main open-reading frames (Figure 1). ORF1 encodes the non-structural polyprotein that includes the enzymes required for HEV replication; these are methyltransferase, which catalyzes the 5′capping of the HEV RNA, helicase, and RNA-dependent RNA polymerase [13]. It is still unclear whether the polyprotein is cleaved during the HEV lifecycle, and if so, whether the HEV cysteine protease is implicated [14]. A recent study has suggested that the structure of the protease is very similar to a fatty acid binding domain that could bind zinc. The binding of fatty acid could regulate the protease activity [15]. Other domains of unknown function have also been described: the Y domain; the polyproline region (PPR), also called the hypervariable region (HVR); and a macro domain, also called the X domain [13]. Sequence analyses suggest that the Y domain is in fact part of methyltransferase [16]. Human gene fragments, such as parts of ribosomal protein genes like S17 [17] or S19 [18], tyrosine amino transferase (TAT), and inter alpha-trypsin inhibitor (ITI) [19], have become inserted into the PPR. Other virus/host recombinant variants have been recently described [20]. Duplications of the HEV genome can also be integrated into the PPR. They can be parts of the genome encoding the PPR ± X domain [19,20], or the one encoding the PPR + RNA polymerase [19,21]. ORF2 encodes the capsid protein. It has recently been shown that the commonly admitted AUG codon in fact encodes a secreted, glycosylated form of ORF2 (ORF2s), while another AUG codon downstream of the first encodes the actual ORF2 capsid protein [22]. Lastly, ORF3 encodes a small protein involved in HEV egress; it acts as an ion channel [23]. Its cysteine residues must be palmitoylated before it can become associated with membranes and facilitate the secretion of infectious particles [24]. HEV1 contains an additional ORF: ORF4, which facilitates HEV replication under stress conditions [25]. HEV is a quasi-enveloped virus that is cloaked in host cell membranes, with no virus glycoproteins in the blood. The bile salts removed these lipids so that the virus excreted in the feces is naked [26,27].

The isolation of HEV strains that replicate efficiently in vitro was very important for establishing cell culture systems. Strains derived from patients’ samples with high HEV loads were used to facilitate initial virus propagation, and thus the production of robust HEV culture systems [28,29]. Infectious complementary DNA (cDNA) clones were then developed: pSK-HEV-2 [30], derived from the HEV1 Sar-55 Pakistani strain, and HEV3-Kernow C1/P6 [31], derived from a chronically infected patient. The Kernow C1/P6 strain replicates efficiently in vitro, due to its incorporation of part of the human gene encoding the S17 ribosomal protein. Incorporation of the same fragment into HEV1 Sar-55 (cDNA clone Sar-55/S17) also enhanced its in vitro fitness [31,32]. Subgenomic cDNA replicon systems have also been developed. The ORF2/3 genes in these systems have been replaced by GFP [33] or a luciferase reporter [34] to monitor replication. Many cell culture systems have also been developed to propagate HEV in vitro [35,36], including hepatoma and non-hepatoma cell lines, stem cell-derived hepatocytes, and primary cells. Polarized systems are a more recent development [37,38]. These systems have been used to study the interaction between HEV and the innate immune system. Animal models, including non-human primates, have also been used to study HEV infection [39,40].

This review summarizes what is presently known of the immune response to HEV, and how the various virus proteins are implicated in immune evasion. The innate immune cell response is not addressed, since it has been recently [41].

## 2. HEV RNA Sensing by Infected Cells

Viral infections usually trigger an innate immune response that results in the production of type I and type III interferons (IFNs) [42]. The first step involves pattern-recognition receptors (PRRs) that recognize molecular structures found in pathogens and named pathogen-associated molecular patterns (PAMPs) [43]. These PAMPs are components usually expressed by the microbial pathogens or generated during infection. Three distinct classes of PRRs that recognize viruses are presently known: toll-like receptors (TLRs) that detect the virus either on cell membranes or in endosomes, retinoic acid-inducible gene I (RIG-I)-like receptors (RLRs), and nucleotide oligomerization domain (NOD)-like receptors (NLRs); both RLRs and NLRs sense virus in the cytoplasm of infected cells [44]. TLRs and RLRs are both expressed by hepatocytes [45]. Once a PRR has recognized a virus, it assembles multiprotein complexes that lead to the production of chemokines and antiviral cytokines, including IFNs. The IFNs trigger the transcription of interferon-stimulated genes (ISGs) that interfere with various steps in virus replication [46].

### 2.1. Recognition by TLR Pattern-Recognition Receptors

**Figure 2:**
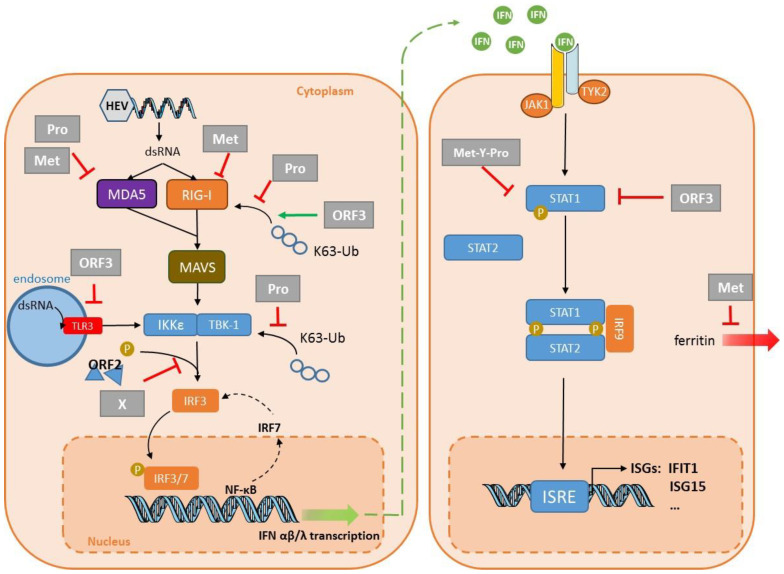
Interplay between innate immunity and hepatitis E virus (HEV). Double-stranded (ds) HEV RNA is detected in the cytoplasm by retinoic acid-inducible gene I (RIG-I) and melanoma differentiation-associated protein 5 (MDA5), leading to type I and type III interferon (IFN) production. TLR3 detects HEV RNA in the endosomal compartment. The protease domain (Pro) of the ORF1 protein inhibits signaling via RIG-I and prevents IFN induction by removing ubiquitin from RIG-I and TANK binding kinase 1 (TBK-1). Pro and Met can also interfere with the MDA5 signaling pathway. The methyltransferase domain (Met) also acts on RIG-I to reduce IFN production, and interferes with ferritin secretion to decrease the inflammatory response. The X domain (X) and the capsid protein ORF2 inhibit the phosphorylation (P) of IFN regulatory protein 3 (IRF3). Conversely, the ORF3 protein stimulates the direct production of type I IFN via RIG-I, while ORF3 interferes with TLR3 synthesis. ORF3 and part of ORF1 (methyltransferase + Y domain + Protease: Met-Y-Pro) also bind to STAT1 to restrict its phosphorylation and the activation of the downstream cascade, thus inhibiting IFN-stimulated gene (ISG) expression, including that of “interferon-induced protein with tetratricopeptide repeats 1” (IFIT1) and ISG15. Abbreviations include IKKε: IκB-kinase-epsilon; IRF3, 7 or 9: IFN regulatory protein 3, 7 or 9; ISRE: interferon-stimulated response element; MAVS: mitochondrial antiviral-signaling protein; STAT1 or 2: signal transducer and activator of transcription 1 or 2; and Ub: ubiquitin.

TLR signaling can take one of two distinct pathways, depending on the adaptor molecules used: all TLRs (except TLR3) use MyD88, while TLR3 and TLR4 use a Toll/IL-1R domain-containing adaptor-inducing IFN- (TRIF) [44]. TLR3 senses double-stranded (ds) RNA (a replication intermediate) in endosomal compartments. Target recognition activates IFN regulatory protein 3 (IRF3) and nuclear factor (NF)-κB via TRIF. IRF3 is then translocated to the nucleus after IKKε /TBK1-mediated phosphorylation. This results in IFN transcription. These IFNs are secreted from the cell and bind to their specific receptors at the cell surface. Type I IFN receptors (IFNARs) are present on all cells; it is composed of two subunits, IFNAR1 and IFNAR2. The type III IFN receptor (IFNLR), preferentially expressed on epithelial cells, is composed of subunit IFNLR1 and a subunit shared by the IL-10 receptor: IL-10Rα. The binding of IFNs to their receptors finally induces hundreds of ISGs, which help inhibit virus replication and spread [47]. We know little about how cells detect HEV and initiate the innate antiviral response. Devhare et al. used several human hepatoma cell lines (Huh7, Huh7.5, and HepG2/C3A) and an HEV1 Sar-55 replicon system to show that the TLR3 signaling pathway restricts HEV replication, suggesting that this TLR works as an HEV RNA sensor [48] (Figure 2). Infection of human lung A549 epithelial cells with an HEV1 strain, derived from a patient’s stools, confirmed the involvement of TLR3 in dsRNA recognition. This study also suggests that TLR2 and TLR4 are involved in the recognition of virus capsid protein [49]. The expression of the TLR3, TLR5, and TLR6 genes in the liver tissue of infected rhesus macaques was downregulated in the early phase of HEV1 Sar-55 and HEV3-JN83748 infections, while TLR3 gene expression was only upregulated at peak infection and declined during HEV3 infection [50].

### 2.2. Recognition by RLR Pattern-Recognition Receptors

The three well-characterized RLRs are RIG-I, melanoma differentiation-associated protein 5 (MDA5), and laboratory of genetics and physiology 2 (LGP2). RLRs are composed of two N-terminal caspase recruitment domains (CARDs), a central DEAD box helicase/ATPase domain, and a C-terminal regulatory domain. LGP2 lacks the CARDs and regulates type I IFN production. All three RLRs are located in the cytoplasm [44]. RIG-I recognizes short dsRNA and 5′-triphosphate RNA, while MDA5 binds to long dsRNA [51]. Ligand-bound RLRs recruit mitochondria-associated antiviral protein (MAVS) to activate transcription factors IRF3/IRF7 and nuclear factor NF-kB; this leads to the production of IFNs and other cytokines (Figure 2). The implication of RIG-I and MDA5 in HEV RNA sensing has been studied in the past few years using transcriptome analysis by TaqMan low-density array (TLDA) or RNA sequencing (RNA seq). The results suggest that these RLRs are important during the infection of human hepatoma cell lines [48,52]. Xu et al. used an HEV3–Kernow replicon system in Huh7.5 cells (a RIG-I-defective hepatoma cell line) to show that the ectopic overexpression of RIG-I delivered by a lentivirus vector strongly reduced HEV3–Kernow replicon luciferase activity 48 h after transduction [53]. IRF1 and MDA5 delivered by the same system also had anti-HEV activity 72 h post-transduction [53]. MDA5 overexpression in Huh7 cells also inhibited HEV3–Kernow replication by triggering an antiviral, IFN-like response [54]. Others have shown that IRF1 has antiviral activity in Huh7 cells transfected with the HEV1–Sar55 or the HEV3–Kernow replicon, as well as in Huh7 cells infected with HEV3–Kernow [55]. Induction of type III IFNs in HepG2 cells infected with HEV3–Kernow depend on MAVS, MDA5, and to a lesser extent RIG-I [56]. However, RIG-I, MDA5, MAVS, or β-catenin knockout cells can still produce IFN in mouse embryonic fibroblast (MEF) cells, suggesting that HEV RNA is recognized by an as-yet-unknown cytosolic RNA sensor, at least in these cells [57]. IFN induction requires IRF3 and IRF7 in this system [57]. These differences in the requirement for RIG-I, MDA-5, and MAVS could be due to the use of different cell lines. The host response of primary human hepatocytes (PHHs) to an HEV3–Kernow infection revealed the intrinsic expression of the pattern recognition receptors RIG-I, MDA-5, and TLR3, as well as downstream signaling molecules, including Myd88 and MAVS, showing that this model can trigger an innate immune signaling cascade [58]. The level of IRF7 mRNA in the livers of rhesus macaques was increased at the peak of HEV1-Sar55 infection [50], but was reduced when the same monkeys were re-infected [59]. IRF3 gene expression was increased at the peak only after infection with HEV3- JN837481 [50]. RIG-I gene expression in the liver tissues from intravenously-infected rhesus macaques was increased at the peaks and declines of both HEV1 Sar-55 and HEV3-JN837481 infections [50], suggesting that it is involved in the anti-HEV response. Thus, both RIG-I and MDA5 are probably implicated in HEV RNA sensing in vitro and in vivo.

### 2.3. HEV Motif Recognized

Wang et al. found that transfection of human embryonic kidney (HEK) 293T cells with vectors expressing ORF2 or ORF3 of HEV3–Kernow had no significant impact on IFN expression, suggesting that these proteins are not sensed by host innate immunity [57]. Conversely, the transfection of Huh7.5 cells with HEV3–Kernow genomic RNA triggered a strong, dose-dependent IFN response, especially IFN-β, IFN-λ1, and IFN-λ2 [57]. Thus, HEV genomic RNA alone is a potent inducer of an antiviral IFN response. Wang et al. also showed that the host response was independent of the capped 5′ terminus or the polyadenylated 3′ terminus. Transfection of HEK293T or Huh7.5 cells with a GAD mutant, a replication-defective HEV3–Kernow replicon in which the aspartic acid in the polymerase active site is replaced with an alanine, resulted in a strong IFN response [57]. This indicates that the antiviral response is independent of HEV replication, which is in line with the response to other viruses, including influenza A virus and respiratory syncytial virus, whose ssRNA stimulates IFN production [60,61]. A recent study demonstrated that the U-rich region at the 3′ end of the HEV genome induced a greater IFN response by hepatoma Huh7 subclone S10-3 cells than did the 5′ untranslated region UTR [62,63]. The same study also confirmed that the loss of the poly-A tail reduces but does not completely abolish the IFN response [62].

## 3. Innate Immune Response to HEV

### 3.1. IFN Response to HEV Infection

When Wu et al. [61] infected induced pluripotent, stem cell-derived, hepatocyte-like cells (iPSC-HLCs) with HEV3–Kernow, they found that IFN-α mRNA remained undetectable, but that IFN-β, -λ1, and -λ3 production increased from day 5. Neither IFN-α nor -β were detected in the supernatant, while the concentrations of type III IFNs (λ1 and λ3) were high [64]. Infecting HepG2 cells and PHHs with the same strain confirmed the increases in type III IFNs λ1 and λ2/3 at the mRNA and protein levels, but not those of type I IFNs [56]. Lastly, gene ontology enrichment analyses of the biological processes of infected PHHs showed that the IFN signaling pathway was among those with the highest ratio of significantly differentially regulated genes [58]. These results are in line with the microarray analyses of the chimpanzees’ liver response to an HEV1–Sar55 infection [65]. IFN-λ3 has also been detected in the sera of patients with an acute HEV infection, suggesting that it plays a role in HEV pathogenesis [66]. The type of IFN produced depends on the nature of the cells infected [62]. The liver tissues of pigs infected with an HEV3–Kernow strain produced a type III IFN response, while the response of infected swine enterocyte IPEC-J2 cells was predominant type I IFN [62]. Primary human intestinal cells infected with clinical HEV1 and HEV3 strains from patients’ stools secreted more type III IFNs (IFN-λ1) than type I IFNs [67]. HEV can replicate in intestinal epithelial cells in the same way as in hepatocytes, despite the presence of a strong IFN- λ1 response [68]. The decidua and placenta produce few type I IFNs (IFN-α2 and IFN-β) [69]. IFN-α2a had no effect on HEV1-Sar55/S17 infections of placenta-derived JEG-3 cells, and produced only a moderate, dose-dependent inhibition of HEV3–Kernow replication [70]. Lastly, HEV1 impaired the production of IFN-λ1 and IFN-λ2/3 by decidual explants and IFN-λ2/3 production by placental explants, while HEV3 had no impact on type III IFN secretion [69].

Activation of IFN receptors triggers the transcription of ISGs. HepG2/C3A cells transfected with capped RNA transcripts from HEV1–Sar55 produced ISGs (ISG15, interferon-induced protein with tetracopeptide repeat 1 (IFIT1, also known as ISG56), IFIT2, etc.) [48]. Similarly, infecting human lung epithelial A49 cells with HEV1–DQ459342 also increased ISG production, including ISG15 or IFIT1 [49]. HepG2 cells or PHHs infected with the HEV3–Kernow strain secreted type III IFNs that activated the transcription of multiple ISGs, including ISG15 and IFIT1 [56,58]. Human liver chimeric mice (homozygous uPA^+/+^-SCID mice) infected with HEV1–Sar55 produced increased IFIT1, while the concentrations of TLR3, ISG15, and MAVS remained unchanged [71]. Microarray analysis of transcriptome profiles also showed significant increases in ISG production in the livers of HEV1–Sar55-infected chimpanzees [65]. An RT 2 profiler PCR array study that compared the host immune responses of rhesus macaques to HEV1–Sar55 and HEV3–JN837481 infections showed that IFIT1, IFIT3, IRF1, IRF7, and ISG15 productions increased, while the synthesis of IFITM1, IFNAR1, IRF2, IRF3, and IRF5 increased only in response to HEV3–JN837481 [50]. Thus, HEV1 and HEV3 trigger different host responses designed to control the viral infection.

### 3.2. Inflammatory Response to a HEV Infection

Little work has been published on the inflammatory response to an HEV infection. A549 cells infected with HEV1–Sar55 produce proinflammatory cytokines/chemokines, including IL-6, TNF-α, and RANTES as early as 12 h post-infection [49]. TNF-α inhibited HEV replication in Huh7 cells transfected with an HEV1–Sar55 replicon by inducing ISGs, including ISG15 and IFIT1 [72]. The anti-viral effect of TNF-α combined with IFN-α on HEV replication in Huh7 cells was additive, due to their cooperation in ISG induction [72]. The inflammatory response to an HEV infection in other tissues in which HEV replicates, such as primary enterocytes, shows that HEV strains induce the secretion of both IL-1α and IL-6, but with different profiles. The HEV3–Kernow strain stimulated more IL-6 production and less IL-1α synthesis than did clinical HEV1 and HEV3 strains from the stools of infected patients [67]. The TNF-α secretions by infected and uninfected cells were similar. Lastly, HEV1 induced markedly more secretion of IL-6 by decidua and placenta tissue explants than did HEV3. However, TNF-α and IL-1 were at best barely detected in these models, regardless of the infection [69]. Animal model studies also confirmed that the inflammatory response was involved in the response to infections with HEV1–Sar55, or human or pig HEV3 strains [50,73].

## 4. Virus Evasion

Unlike HAV [74] and HCV [75], HEV protease does not cleave MAVS [56]. Nevertheless, HEV has developed many other strategies for interfering with the innate immune response: it can disrupt the IFN response by inhibiting its production, or by limiting its effect once it has interacted with its receptor (Table 1).

The ORF1 polyprotein interferes with the establishment of the IFN response by acting on several targets (Figure 2). Nan et al. showed that HEV1–Sar55 ORF1 protein inhibits type I IFN production by HEK293T cells by inhibiting RIG-I signaling [76]. The protease removes the ubiquitin from both RIG-I and TBK-1, thus impairing the signaling pathway leading to IFN production [76]. The reduced RIG-I and TBK-1 ubiquitination was confirmed with a replicon system in Huh7 S10-3 cells. The lack of IFN response gene expression by rhesus macaques infected with HEV1–Sar55 [50] is in accord with the ability of the HEV1 ORF1 polyprotein to block PRR signaling [76]. The protease and the methyltransferase inhibited the MDA5-induced activation of IFN-β induction [77,78]. The methyltransferase inhibited the MDA5-mediated phosphorylation of the p65 subunit of NF-kB, but this effect seemed strain-dependent [79]. This group used the same system to show that the methyltransferase dose-dependently decreased RIG-I-induced IFN-β induction [80]. Whether the methyltransferase targets RIG-I directly or indirectly remains unclear. The X domain of HEV1–Sar55 impairs the phosphorylation of IRF3 in the hepatoma Huh7 S10-3 cell line, leading to decreased IFN production [76]. The X domain of HEV1–Sar55 interacts with the light chain of human ferritin, an acute phase protein, in Huh7 S10-3 cells transfected with a plasmid encoding this domain [81]. The authors of this study proposed that this interaction could prevent ferritin secretion and therefore restrain the innate immune response. These interesting results should perhaps be interpreted cautiously, since transfection is very efficient in HEK293T; they need confirmation using infected hepatoma cell lines or PHHs.

Infected cells can produce IFNs in spite of these mechanisms designed to counteract IFN synthesis. Consequently, HEV has developed ways to impair the IFN signaling pathway, and to reduce the effects of the ISGs. ISG15 is a small, ubiquitin-like molecule that has many roles [82]. It can have a direct antiviral effect on viruses like HIV [83] and HCV [84]. Conversely, it may promote HCV replication [85]. In HEV infections, ISG15 acts as an immunomodulator, favoring HEV replication. Silencing the ISG15 gene in Huh7 S10-3 cells transfected with HEV3–Kernow RNA transcripts enhanced the antiviral effect mediated by type I IFNs [86]. The methyltransferase–protease domain of HEV1–DQ459342 hydrolyses ISG15-conjugated cellular proteins, but whether this deISGylation activity is required to enhance HEV replication remains to be determined [87]. Bagdassarian et al. showed that HEV3–MG197988 polyprotein interferes with IFN signaling in HEK293T cells. The three domains—methyltransferase, Y, and protease (Met-Y-Pro)—are needed to inhibit the signaling pathway triggered by type I IFNs. They seem to interfere with the phosphorylation of STAT1 and its translocation to the nucleus after IFN-β treatment [88] (Figure 2). Although the amino acid sequences of the Met-Y-Pro domains of HEV1 and HEV3 are 86% identical, HEV1 Met-Y-Pro does not inhibit the JAK/STAT pathway as efficiently as that of HEV3 [88]. IFIT1 regulates the translation of foreign or non-self RNAs by recognizing the mRNA whose ribose 2′-OH group is unmethylated (cap0); this structure is common in most viral mRNAs [89]. IFIT1 also binds to cap0 mRNA or mRNA with a free 5′-triphosphate group [90]. It was recently proposed that IFIT1 was sequestered by the HEV1–Sar55 RdRp to inhibit its anti-translational activity, at least in Huh7 S10-3 cells [91].

ORF2 protein from HEV1–Sar55 and HEV3–Kernow could inhibit IFN production in HEK293T cells by blocking the phosphorylation of IRF3 [92] (Figure 2). Thus, ORF2 interacts directly with the MAVS–TBK1–IRF3 complex to inhibit the phosphorylation of IRF3 and its dissociation from the complex. The interaction of ORF2 with TBK1 was confirmed by co-immunoprecipitation in HepG2/C3A cells infected with HEV3–Kernow [92]. Others have confirmed that the ORF2 protein from HEV1–Sar55 and HEV3–Kernow inhibits IFN-β production in HEK293T cells by interfering with TLR and RIG-I signaling [93]. The molecular target has not yet been clearly identified, but it is downstream from the adapter proteins and upstream of IRF3. Lastly, ORF2 from HEV1-Sar55 impairs the Huh7 apoptotic mechanisms, enabling the virus to complete its lifecycle [94].

The ORF3 of HEV1-Sar55 inhibits the production of type I IFNs in human monocytic THP1 and human hepatic Lo2 cells by inhibiting TLR3 and TLR7 expression [97]. Another group has reported that HEV1–Sar55 ORF3 inhibits the TLR3-mediated NF-κB activity in A549 cells [96]. In contrast, the ORF3s of HEV1–Sar55 and HEV3–Kernow enhance the activation of the IFN-β promoter in HEK293T cells [95]. They do so by stimulating polyubiquitination, and hence the activation of RIG-I (Figure 2). However, HEV3–JN837481 ORF3 inhibits the IFN-α-induced phosphorylation of STAT1 in A549 cells [98], thus reducing ISG production. These apparently contradictory observations were rendered compatible by a recent study. The transfection of capped RNA transcripts of the HEV3–Kernow strain without the S17 fragment first enhanced IFN-α/β production, and then that of ISG15 in HepG2/C3A cells. The increase in ISG15 in turn reduced the IFN concentrations [99]. Lastly, Wang et al. suggested that ORF3 is responsible for enhancing ISG15 synthesis, since virus lacking ORF3 triggered the production of less ISG15 [99]. The exact role of ORF3 in immune evasion needs to be confirmed in infection conditions using PHHs.

No study has yet been performed to determine whether ORF4 interferes with the establishment of the innate immune response.

## 5. Conclusions

Many studies have documented the capacity of virus proteins to interfere with the innate immune response. HEV proteins can interfere with the IFN system by inhibiting the signaling cascades, leading to the activation of IFN genes or disrupting the IFN signaling pathway that activates the transcription of ISGs. The most recent studies have focused on the role of MDA5 and how HEV disrupts MDA5 receptor signaling pathway. Although these results are interesting and important, many of these studies were performed using overexpression systems and recombinant strains. Whether the results obtained with recombinant strains reflect the behavior of wild-type HEV needs to be confirmed with clinical strains that have not been adapted to cell culture systems [100]. Studies using relevant in vitro culture systems, especially PHH, are also essential to confirm these results. Recently developed new cell culture systems and animal models will facilitate confirmation of these observations.

## Figures and Tables

**Table 1:** The main mechanisms of HEV escape.

Target	Cell Line	Strain	System	Key Finding	Reference
PRR signaling	HEK293T Huh7 S10-3	HEV1–Sar55	Transfection: plasmid encoding the domains of ORF1 Replicon	The protease can de-ubiquitinate RIG-I and TBK-1. Confirmed in a replicon system and S10-3 cells.	Nan, 2014 [76]
	HEK293T	HEV3–47832c	Transfection: plasmid encoding Pro	The protease inhibits IFN-β induction by interfering with MDA5 signaling.	Kim, 2018 [77]
	HEK293T	HEV3–47832c	Transfection: plasmid encoding Met	The methyltransferase inhibits IFN-β induction by interfering with RIG-I signaling.	Kang, 2018 [80]
	HEK293T	HEV3–47832c	Transfection: plasmid encoding Met	The methyltransferase inhibits IFN-β induction by interfering with MDA5 signaling.	Myoung, 2019 [78]
	HEK293T	HEV3–47832c	Transfection: plasmid encoding Met	The methyltransferase inhibits IFN-β induction by inhibiting MDA5-mediated phosphorylation of NF-κB.	Myoung, 2019 [79]
	HEK293T Huh7 S10-3	HEV1–Sar55	Transfection: plasmid encoding the domains of ORF1 Replicon	The X domain impairs IRF3 phosphorylation. Confirmed in a replicon system in S10-3 cells.	Nan, 2014 [76]
	HEK293T HepG2/C3A	HEV1 Sar55 HEV3–kernow	Transfection: plasmid encoding ORF2 (HEV1/3) plus HEV3 infection	ORF2 interacts with TBK1 to impair IRF3 phosphorylation and its dissociation from MAVS.	Lin, 2019 [92]
	HEK293T Huh7	HEV1–Sar5 HEV3–Kernow	Transfection: plasmid encoding the ORF2 protein	ORF2 inhibits IFN production by blocking TLR and RIG-I signaling pathways.	Hingane, 2020 [93]
	HEK293T	HEV1–Sar55 HEV3–Kernow	Transfection: plasmid encoding the ORF3 protein	ORF3 from HEV1 and HEV3 interact with RIG-I to increase its ubiquitination.	Nan, 2014 [95]
	A549	HEV1–Sar55	Transfection: plasmid encoding the ORF3 protein	ORF3 blocks TLR3-mediated NF-κB activity.	He, 2016 [96]
	THP1 Lo2	HEV1–Sar55	Transfection: plasmid encoding the ORF3 protein	ORF3 reduces TLR3 and TLR7 expression in these two cell lines.	Lei, 2018 [97]
IFN signaling	A549	HEV3–JN837481	Infection	HEV ORF3 protein blocks IFN-α-induced STAT1 phosphorylation and impairs IFNα-induced gene expression.	Dong, 2012 [98]
	HEK293T	HEV3–MG197988	Transfection: plasmid encoding Met-Y–Pro	Met-Y-Pro domain interferes with STAT1 phosphorylation and subsequent nuclear translocation. HEV1 Met-Y-Pro domain interferes less efficiently than HEV3 Met-Y-Pro domain.	Bagdassarian, 2018 [88]
Interferon Stimulated Genes	in vitro and HepG2	HEV1–DQ459342	Transfection: plasmid encoding the Met Pro domain	The protease domain has a deISGylation activity.	Karpe,2011 [87]
	Huh7 S10-3	HEV3–Kernow	Transfection: in vitro capped RNA transcript replicons	HEV induces ISG15 in vitro and in liver tissues of infected pigs. ISG15 is immunomodulatory—enhances HEV replication.	Sooryanarain, 2017 [86]
	HepG2/C3A	HEV3–Kernow without S17 fragment	Transfection: in vitro capped RNA transcripts Transfection: plasmid encoding the ORF3 protein	ORF3 enhances ISG15 production, hence HEV replication.	Wang, 2018 [99]
	Huh7 S10-3	HEV1–Sar55	Transfection: plasmid encoding RdRp	RdRp sequesters IFIT1 to inhibit its anti-translational activity.	Pingale, 2019 [91]
Other	Huh7 S10-3	HEV1–Sar55	Transfection: plasmid encoding X-domain replicon	X domain interacts with the light chain to prevent its secretion, restraining innate immunity.	Ojha, 2016 [81]
	Huh7	HEV1–Sar55	Transfection: plasmid encoding the ORF2 protein	ORF2 impairs apoptosis, allowing HEV lifecycle completion.	John, 2011 [94]
